# Global Prevalence and Cancer Risk of Epstein–Barr Virus and Human Papillomavirus Coinfection in Breast Cancer: A Systematic Review and Meta-Analysis

**DOI:** 10.3390/v17121592

**Published:** 2025-12-08

**Authors:** Abdelrahman A. Karen, Albara S. Elkhalaf, Omar Tluli, Omar Sorour, Abdulnaser Fakhrou, Mohammed Imad Malki, Karim Nagi

**Affiliations:** 1 Department of Basic Medical Sciences, College of Medicine, QU Health, Qatar University, Doha P.O. Box 2713, Qatar; ak2104882@student.qu.edu.qa (A.A.K.); ae2104790@student.qu.edu.qa (A.S.E.); ot2004691@student.qu.edu.qa (O.T.); oa2107382@student.qu.edu.qa (O.S.); momalki@qu.edu.qa (M.I.M.); 2 Psychological Sciences, College of Education, Qatar University, Doha P.O. Box 2713, Qatar; afakhrou@qu.edu.qa

**Keywords:** breast cancer, Epstein–Barr virus (EBV), human papillomavirus (HPV), prevalence, oncoviruses, meta-analysis

## Abstract

Background: Breast cancer (BC) is the most frequently diagnosed malignancy and a dominant cause of cancer mortality among women worldwide. Alongside established risk factors, recent studies highlight oncoviruses like Epstein–Barr virus (EBV) and human papillomavirus (HPV) as potential contributors. However, their role and association with BC development is still debatable. Study design and Methods: This systematic review and meta-analysis involved two distinct approaches: one assessing the worldwide prevalence of EBV and HPV coinfection in BC patients and another investigating the association between such coinfection and BC risk. A systematic search across PubMed, Scopus, Web of Science, and Embase was conducted up to 5 May 2025. Studies using PCR to detect both viruses in breast tissue samples were included. Random-effects models were used to estimate pooled prevalence and odds ratios with 95% confidence intervals. Results: Out of 307 non-duplicate records, 16 studies were found to be eligible for quantitative analysis. The pooled prevalence of EBV/HPV coinfection among BC patients was 14% (95% CI: 12–16%; I^2^ = 91.0%). Prevalence varied by region, ranging from 6% in South America to 22% in the Middle East. In addition, a general trend towards increasing EBV/HPV coinfection prevalence among women with BC over time was detected. Moreover, analyzing case–control studies to investigate the relationship between EBV/HPV coinfection and the risk of BC, the pooled odds ratio was 5.87 (95% CI: 2.31–14.93; I^2^ = 0%, *p* = 0.91). Conclusion: Our analysis shows that EBV and HPV coinfection prevalence varies by region and appears to be rising over time among women with breast cancer. Additionally, the strong statistical association between coinfection and breast cancer risk suggests a potential role for these oncoviruses in disease development, highlighting the possible preventive value of EBV and HPV vaccination.

## 1. Introduction

Breast cancer (BC) is the most frequently diagnosed cancer among women globally and remains the primary cause of cancer-related mortality in females. In 2022 alone, approximately 2.3 million women were diagnosed with breast cancer, representing 23.8% of all new cancer cases and 15.4% of all cancer-related deaths among women worldwide [1]. Despite significant advances in understanding its biological and molecular characteristics, the etiology of BC remains multifactorial and incompletely understood. Established risk factors such as genetic mutations in BRCA1 and BRCA2, hormonal exposure, reproductive history, and lifestyle choices are well documented [2]. However, emerging evidence suggests that infectious agents, particularly oncogenic viruses, may also contribute to breast carcinogenesis in ways that are still being explored [3]. Among the viral candidates, Epstein–Barr virus (EBV) and human papillomavirus (HPV) have garnered the most attention, particularly for their possible synergistic effects on cellular transformation and tumor progression.

A growing number of studies have proposed that coinfection with EBV and HPV may exert a synergistic oncogenic effect, enhancing tumor initiation and progression more effectively than either virus alone [4,5]. This dual infection can potentiate cellular transformation by enhancing inflammatory signaling, disrupting epithelial integrity, and prolonging viral persistence within the host tissues [6,7]. More precisely, HPV oncoproteins E6 and E7 inactivate tumor suppressors p53 and pRb, inducing genomic instability and uncontrolled proliferation [8], while EBV latent proteins LMP1, LMP2A, and EBNA1 activate NF-κB, JAK/STAT, and PI3K/Akt pathways that promote cell survival, angiogenesis, and immune evasion [9,10,11]. Co-expression of these viral proteins amplifies proliferative and anti-apoptotic signaling, increases oxidative stress, and in-duces epithelial–mesenchymal transition via Snail and Twist upregulation [12,13]. Furthermore, both viruses impair antigen presentation and modulate cytokine signaling, thereby promoting persistent infection and chronic inflammation that collectively establish a microenvironment favorable to tumor progression [10]. These combined effects foster a tumor-permissive niche that facilitates the malignant transformation and progression of breast epithelial cells. This raises the possibility that viral detection could one day become a meaningful component of clinical evaluation and risk stratification in breast cancer care.

This systematic review and meta-analysis was therefore conducted to evaluate the global prevalence and cancer risk of EBV and HPV coinfection in BC patients. Only studies using polymerase chain reaction (PCR) for viral detection were included to ensure methodological consistency and improve the reliability of prevalence estimates. By synthesizing findings from diverse populations and study designs, this analysis aims to clarify the epidemiological landscape of viral coinfection in BC and establish a foundation for future research exploring its clinical relevance. Moreover, such insights could pave the way for novel therapeutic or preventive strategies, including the use of antiviral agents or virus-targeted vaccines for patients with virus-associated tumors [14]. Identifying consistent patterns in coinfection prevalence may inform future efforts to standardize diagnostic protocols, investigate the mechanistic role of viral synergy, and assess the potential benefit of incorporating viral screening into BC care models.

## 2. Materials and Methods

### 2.1. Data Sources and Search Strategy

**Figure 1:**
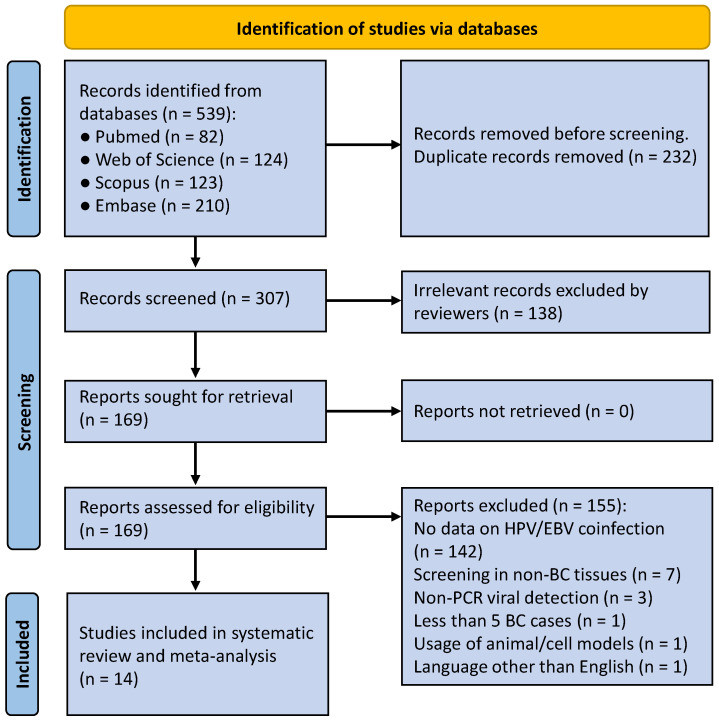
PRISMA flow chart for studies selection.

A systematic search strategy was conducted in compliance with the Preferred Reporting Items for Systematic Reviews and Meta-Analysis (PRISMA) guidelines (see the PRISMA checklist in the Supplementary Materials, Table S1) to identify studies reporting the prevalence of EBV and HPV coinfection in patients with BC (Figure 1). The search was performed from inception to 5 May 2025 across four major databases: PubMed, Scopus, Web of Science and Embase. The full search strategy used for each database is presented in Appendix A. The meta-analysis protocol was submitted to the international prospective register of systematic reviews (PROSPERO) online database (PROSPERO Identifier: CRD420251233583).

### 2.2. Eligibility Criteria

Studies that evaluated the prevalence of EBV and HPV coinfection in patients diagnosed with BC were included in this systematic review. Databases were searched without any date or publication restriction. However, studies that examined the presence of oncoviruses in samples other than breast tissue, such as milk or blood; used methods other than PCR for DNA detection or assessed fewer than five cases were excluded. In addition, non-English and non-human (animal, cell lines) studies were also excluded. A comprehensive summary of the excluded studies and reasons for their exclusion can be found in Table S2.

### 2.3. Study Screening and Selection

Two independent investigators (A.A.K., A.S.E.) conducted a search of articles across the different databases and removed any duplicates. An initial screening of titles and abstracts was then performed to identify studies that met the inclusion criteria. Afterwards, full-texts of the selected articles were reviewed to exclude irrelevant publications. Any disagreement during this process was settled through discussion with a third investigator (K.N.).

### 2.4. Data Extraction

Characteristics for all eligible studies were recorded in a data extraction spreadsheet. The following information were extracted from each article: first author name, year of publication, country of origin of the population group, clinical profile of BC patients, total number of cases, number of BC samples included in analysis and reasons for exclusion, number and percentage of positives in the case and control groups, mean patients age, sample type, detection method, most prevalent HPV types in EBV positive cases, and the study type (Tables S3 and S4). For each study, the reported odds ratios (ORs) represented the association between EBV and HPV coinfection and breast cancer risk relative to virus-negative controls. Data extraction was performed by three reviewers independently, with discrepancies resolved by consensus. All data have been compiled and are provided in the Supplementary Materials (Tables S3 and S4).

### 2.5. Quality Assessment

The quality of included studies was evaluated using the Joanna Briggs Institute (JBI) critical appraisal tool specifically designed for case–control and cross-sectional studies. These quality assessment tools were employed to evaluate the methodological quality and to determine how effectively any potential bias in the study’s design, conduct, and analysis was addressed. Each of these domains was assessed using designed questions that could be answered with a ‘Yes’ (Y), ‘No’ (N), ‘Unclear’ (UC) or ‘Not applicable’ (NA). Each study was given a final quality score. However, when certain JBI criteria were deemed not applicable to a study, they were excluded from the denominator when calculating the final quality score, ensuring that the overall quality rating reflected only the criteria relevant to each study design. Studies were categorized as high quality (above 80%), moderate (60–80%) or low quality (below 60%) studies. OT and OA independently evaluated the quality of the included studies, and any disagreements were resolved through discussion with a third investigator. Results of the quality assessment scoring are presented in the Supplementary Materials (Table S5).

### 2.6. Statistical Analysis

The present meta-analysis involved two distinct approaches: one assessing the prevalence of EBV and HPV coinfection in BC patients and another investigating the association between EBV/HPV coinfection and BC risk. Two key parameters were primarily considered: the prevalence rate of EBV and HPV coinfection and the odds ratio for the association between EBV/HPV coinfection and BC risk. For the prevalence analysis, the proportion of EBV and HPV coinfection was pooled in each country, where sufficient data were available, and using the inverse variance method with a random-effects model. The Clopper-Pearson method was used to calculate the confidence intervals (CIs) for individual study proportions [15]. In studies with zero detection of oncoviruses coinfection among BC cases, a continuity correction of 0.5 was applied. For the association between EBV/HPV coinfection and BC risk, pooled odds ratios with 95% CIs were calculated using the random-effects model. The Z-test was employed to assess the statistical significance of the pooled ORs, with a significance threshold set at *p* < 0.05. Heterogeneity across studies was evaluated using both the Mantel–Haenszel and DerSimonian–Laird methods and was quantified using the I^2^ test, where statistical significance for heterogeneity was set at a p-value <0.10. All analyses were conducted using Review Manager 5.4. Publication bias was evaluated using both funnel plot and Egger’s regression test. A funnel plot of the log-transformed odds ratios against their standard errors was generated to visually assess potential asymmetry. Egger’s regression test was performed to statistically detect the presence of small-study effects. A *p*-value below 0.05 was considered indicative of potential publication bias. Both funnel plot and Egger’s regression test were conducted using MetaAnalysisOnline tool. Sensitivity analyses were performed to assess the robustness of pooled estimates. Subgroup analysis compared studies using PCR alone with those employing combined or alternative EBV detection methods to assess methodological variability. Another subgroup analysis stratifying studies by E6/E7 and L1 regions was performed to assess the influence of viral gene targets on pooled prevalence. Separately, studies rated as low quality were excluded to examine the impact of study quality on prevalence and heterogeneity. Also, to evaluate the impact of methodological variability among included studies, we conducted a sensitivity analysis in which the meta-analysis results were re-weighted according to study quality. Study quality scores were derived from the JBI critical appraisal tool (Table S5). The resulting pooled estimate was compared with the random-effects model to assess robustness. Consistent results across these analyses confirmed the stability of the pooled findings.

## 3. Results

### 3.1. Study Selection

We initially identified 539 papers from the four databases. After the removal of duplicates, 307 papers were remained for screening the title and abstract. Of these, 291 were excluded based on the defined criteria. The reasons for exclusion are detailed in methods section (Section 2) and summarized in Table S2. Finally, a total of 14 data sources were finally included in the final analysis.

### 3.2. Characteristics of Included Data Sources

The characteristics of eligible studies included in this systematic review and meta-analysis are summarized in Table S3. Among the 14 included studies, 10 had case–control design and 4 were cross-sectional. Studies were published between 2011 and 2024 and collectively included a total of 1679 woman diagnosed with BC spanning 13 countries across six regions. However, some samples were excluded due to negative results in the beta-globin test and incomplete data from PCR or IHC analyses, resulting in a final total of 1180 eligible samples for final analysis. The majority of studies originated from the Middle East (6 studies), followed by Asia (2), South America (2), North Africa (2), and one study each from Europe and Australia. Three studies analyzed samples from woman diagnosed with BC prior to 2012 [16,17,18,19,20], while six studies examined samples from patients recruited between 2012 and 2022 [21,22,23,24,25,26]. One study analyzed samples from woman with BC treated surgically during 2008–2019 [27] and another during 2006–2016 [28] (Table S3).

### 3.3. Risk of Bias Assessment of Included Studies

To ensure a rigorous evaluation of risk of bias across all included studies, the JBI critical appraisal tools were employed, with each tool tailored to the specific study design identified in the systematic review. The quality of case–control [18,21,22,23,24,25,26,28,29,30] and cross-sectional [16,17,27,31] studies was assessed using the corresponding JBI checklists designed for each study type. Results of the quality assessment checklists are presented in Table S5. The quality of the included studies varied across evaluated domains (Table S5A,B). Most studies were of moderate to high quality, with well-defined methodology, clear inclusion criteria, and appropriate outcome assessment. One study was rated as low quality due to incomplete reporting of the inclusion criteria and limited control for confounders factors. A sensitivity analysis excluding this low-quality study (Figure S1) yielded a pooled prevalence of 12.4% (95% CI: 10–15%), compared with 14.2% (95% CI: 12–16%) when all studies were included. This difference was not statistically significant (z = 1.39, *p* = 0.17), indicating the robustness of the overall findings. Thus, this study was retained in the final analysis to preserve the comprehensiveness of the evidence base, as its exclusion did not influence the robustness or direction of the overall findings.

### 3.4. Prevalence of EBV/HPV Coinfection Among Patients with BC

**Figure 2:**
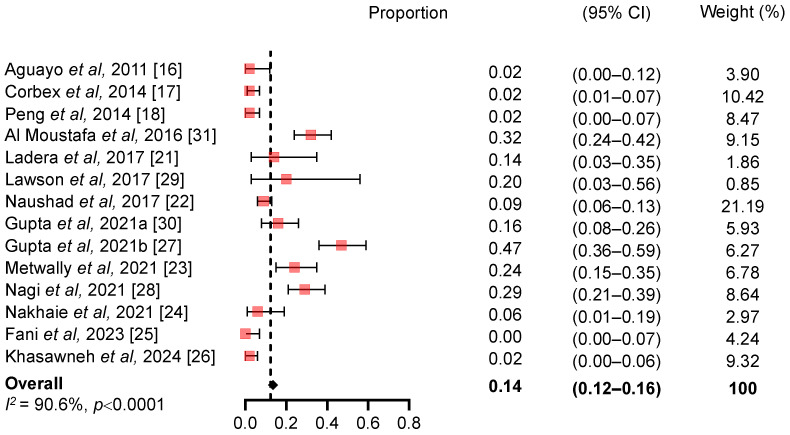
Forest plot of the prevalence of human papillomavirus and Epstein–Barr virus coinfection among patients with breast cancer. References [16,17,18,21,22,23,24,25,26,27,28,29,30,31] are cited in this figure.

The raw prevalence of EBV/HPV coinfection in women with BC varied widely across studies (Figure 2). Three studies reported a prevalence of 0%: two conducted between 2001 and 2011 [19] and one between 2018 and 2021 [25]. In contrast, the highest prevalence (47%) was observed in a study from Qatar covering the period 2008–2019 [27]. Notably, only two studies were conducted in the same country (Iran), with coinfection prevalence ranging from 0% (95% CI: 0–0.07) in 2018–2021 [25] to 6% (95% CI: 0.01–0.19) in 2020 [24]. The overall pooled prevalence of EBV/HPV coinfection from all the 14 studies was 14.2% (95% CI: 12–16%) with high between-study heterogeneity (I^2^ = 90.6%, *p* < 0.0001) (Figure 2). Detection of viruses in tumor tissues is highly sensitive to the method used and employing different techniques and protocols could introduce between-study heterogeneity. We carefully documented the detection methods applied in all studies that identified the presence of both HPV and EBV viruses in breast related tissues in order to provide a comprehensive overview of the literature (see Table S6). All studies investigating HPV and EBV co-infection in breast cancer (BC) samples utilized paraffin-embedded tissue blocks from BC patients. Notably, all these studies, used PCR to detect HPV. Therefore, comparing PCR-based studies with those using alternative viral detection methods in BC samples was not feasible. Methodological variability was mainly related to the approaches used for EBV detection. Most studies relied on PCR alone, while others combined PCR with additional techniques, including three with in situ hybridization (ISH), one with reverse hybridization, two with immunohistochemistry (IHC), one with both IHC and ISH, one with IHC and next-generation sequencing (NGS), and one study that used ISH exclusively. Given this methodological heterogeneity, we conducted a sensitivity analysis (see Figure S2) comparing the pooled prevalence obtained from studies using PCR alone with those employing alternative or combined methods for EBV detection. In this analysis, studies using PCR exclusively for EBV detection reported a pooled prevalence of 13.8% (95% CI: 11–17%; I^2^ = 91.6%, *p* < 0.0001), which was comparable to that of studies combining PCR with other detection techniques such as IHC or ISH (14.8%, 95% CI: 12–18%; I^2^ = 91.1%, *p* < 0.0001) (Figure S2A,B). In contrast, studies using non-PCR-based approaches (e.g., IHC, ISH, or NGS) reported no EBV detection (0%, 95% CI: 0–3%; I^2^ = 0%, *p* = 1.0) (Figure S2C). These results suggest that PCR-based techniques might offer a superior sensitivity for detecting EBV in breast cancer tissues, likely due to their ability to detect low-copy viral genomes even in the absence of active transcription or protein expression.

We performed a second subgroup analysis based on viral gene targets to account for potential differences in detection sensitivity and specificity (Figure S3). Notably, all the included studies targeted the EBNA1/EBNA2 region of EBV, ensuring a methodological consistency across studies. The main variation in gene targets was observed in HPV detection; therefore, studies were stratified into two subgroups based on PCR assays targeting either the early (E6/E7) or late (L1) regions of HPV (Figure S3) to account for potential differences in detection sensitivity and specificity. Seven studies targeted E6/E7 and six targeted L1; only one study targeted both E6/E7 and L1 regions and was excluded from the sensitivity analysis. Comparison revealed that E6/E7 prevalence was significantly higher than L1 prevalence (Z = 3.75, *p* < 0.001) and higher than the overall pooled prevalence (Z = 3.41, *p* < 0.001), suggesting a greater assay sensitivity of early region targets. In contrast, L1 prevalence did not differ significantly from the overall estimate (Z = –1.65, *p* = 0.099). Together, these findings indicate that PCR assays targeting the E6/E7 region showed higher detection rates than those targeting the L1 region, underscoring the influence of genomic region selection on methodological heterogeneity and reinforcing the importance of gene-target selection when interpreting pooled prevalence outcomes.

### 3.5. Prevalence of EBV/HPV Coinfection in BC Women by Region

**Figure 3:**
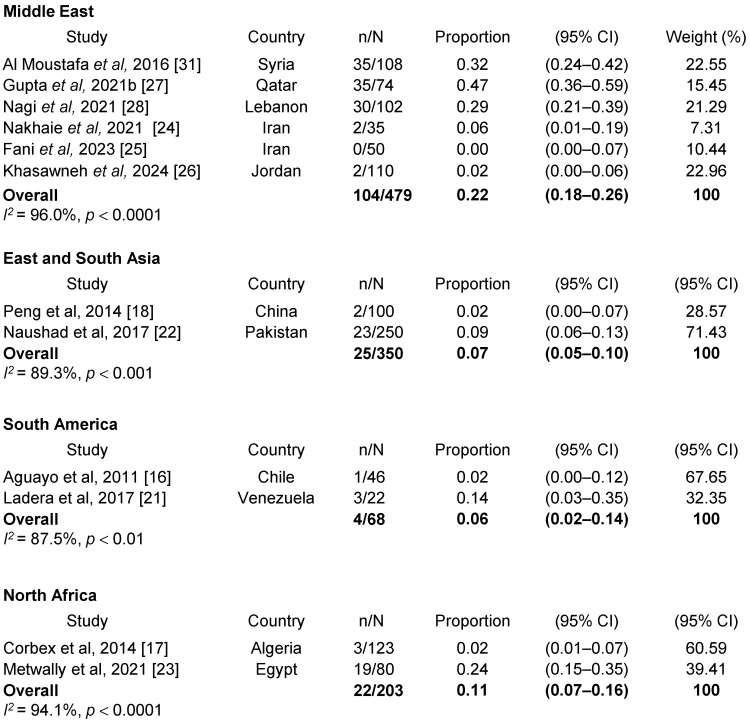
Prevalence of EBV/HPV coinfection in BC women by region. References [16,17,18,21,22,23,24,25,26,27,28,31] are cited in this figure.

The pooled prevalence showed regional variation, with the lowest prevalence reported in South America (6%, 95% CI: 2–14%; I^2^ = 87.5%, *p* < 0.01) and the highest prevalence in the Middle East region (22%, 95% CI: 18–26%; I^2^ = 96.0%, *p* < 0.0001) (Figure 3). South America and East/South Asia displayed similar pooled prevalence rates, ranging between 6% and 7%. In the Middle East, prevalence varied from 0% in a study from Iran [25] to 47% in a study from Qatar [27]. All studies from East and South Asia, South America, and North Africa regions reported prevalence above 2%, with the highest prevalence being 11% (95% CI: 7–16%; I^2^ = 94.1%, *p* < 0.0001) in North Africa. In one study from Egypt, 19 out of 80 women diagnosed with BC were co-infected with both EBV and HPV. Notably, one study from Egypt reported that 19 women out of 80 women diagnosed with BC were coinfected with EBV and HPV viruses. In Europe and Australia, only one study from each region was available for inclusion, with reported prevalence of 16% (95% CI: 8–26%) in Croatia [30], and 20% (95% CI: 3–56%) in Australia [29].

### 3.6. Prevalence of EBV/HPV Coinfection in BC Women by Study Period

**Figure 4:**
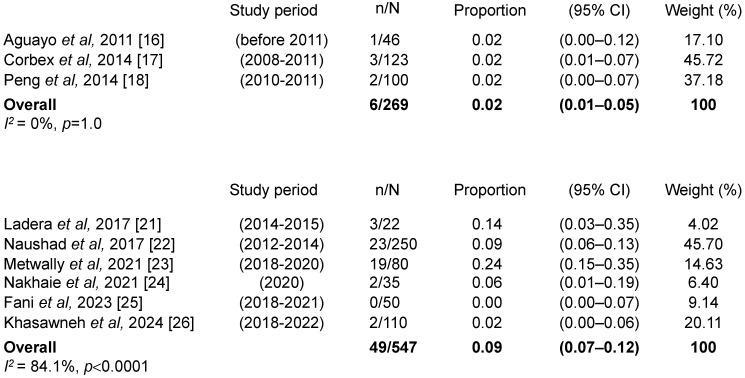
Prevalence of EBV/HPV coinfection in BC women by study period. References [16,17,18,21,22,23,24,25,26] are cited in this figure.

Studies were grouped based on the time period during which female patients were diagnosed with BC and sample collection was performed. Overall, there was a general trend towards increasing EBV/HPV coinfection prevalence among women with BC over time, from 2% (95% CI: 1–4%; I^2^ = 0%, *p* = 1.0) during the period preceding 2012 to 9% (95% CI: 7–12%; I^2^ = 84.1%, *p* < 0.0001) during the period 2012–2024 (Figure 4). Additionally, two studies spanning broad timeframes demonstrated a similar upward trend. Notably, one study that analyzed samples from Lebanese women treated surgically between 2006 and 2016 [28], reported a prevalence of 29% (95% CI: 21–39%), while another study conducted over a later time period using samples from Qatari women treated surgically between 2008 and 2019 [27], found a higher prevalence of 47% (95% CI: 36–59%).

### 3.7. Association Between EBV/HPV Coinfection and BC

**Figure 5:**
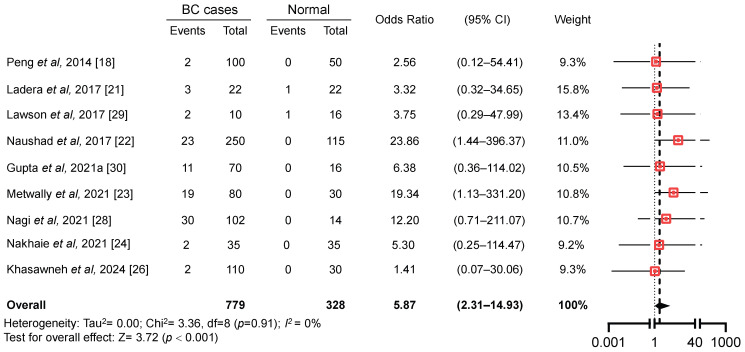
Forest plot of the association between human papillomavirus and Epstein–Barr virus coinfection and breast cancer risk worldwide [18,21,22,23,24,26,28,29,30].

The next phase involved conducting a second meta-analysis using case–control studies to investigate the relationship between EBV/HPV coinfection and the risk of BC (Figure 5). Out of ten eligible studies with case–control design, only one study was excluded due to the absence of EBV/HPV coinfection in both the case and control groups [25], resulting in nine studies being included in the final analysis. These studies collectively included 771 BC cases and 294 controls. However, due to absence of beta-globin testing or missing molecular subtyping data, some samples were excluded, yielding a final dataset of 744 BC and 293 control samples eligible for final analysis. The pooled odds ratios reflect the relative risk of breast cancer in individuals coinfected with EBV and HPV compared with virus-negative controls. Only a few of the included studies provided sufficient information to allow separate quantitative comparisons between coinfected and singly infected groups (EBV-only or HPV-only). Therefore, the present analysis focuses on the overall oncogenic risk attributable to dual infection, acknowledging that assessment of additive or synergistic interactions requires further primary data.

Using a random-effects model, the pooled odds ratio for the association between EBV/HPV coinfection and BC risk was 5.87 (95% CI: 2.31–14.93; Z = 3.72; *p <* 0.001; I^2^ = 0%) (Figure 5). This indicates a strong and statistically significant association, with individuals coinfected with EBV and HPV having 5.87 times higher odds of developing BC compared to those not coinfected with these oncoviruses. The absence of heterogeneity (I^2^ = 0%) further reinforces the reliability and consistency of these findings across the included studies. Moreover, the funnel plot displayed a largely symmetrical distribution of studies around the pooled estimate, indicating no apparent publication bias (Figure S4). Consistently, Egger’s regression test showed no significant evidence of small-study effects (intercept = 0.46, 95% CI: −4.69–5.62%; *t* = 0.177; *p* = 0.865). Additionally, to evaluate the impact of methodological variability among the included studies, we performed a sensitivity analysis by weighting the meta-analysis results according to study quality. Study quality scores were derived from the JBI critical appraisal tool (Table S5). Results derived from this sensitivity analysis (Figure S5) showed an overall odds ratio of 5.86 (95% CI: 2.31–14.88), which was nearly identical to the overall odds ratio derived from the random effects model (5.87; 95% CI: 2.31–14.93). These results collectively suggest that the pooled odds ratio is robust and unlikely to be influenced by potential publication bias or by differences in study quality.

## 4. Discussion

The relationship between oncogenic viruses and human cancer development has been a subject of extensive scientific inquiry. Among the numerous viruses implicated in carcinogenesis, EBV and HPV stand out due to their established roles in various epithelial and lymphoid malignancies [32,33]. While previous meta-analyses have explored the individual associations of EBV or HPV infection with breast cancer risk, the potential synergistic oncogenic impact of coinfection has not been comprehensively evaluated. The present study thus complements prior evidence by focusing on the combined viral effect relative to virus-negative controls. To our knowledge, this is the first systematic review and meta-analysis to investigate EBV/HPV coinfection, rather than assessing each virus independently in BC patients. Moreover, previous studies have produced conflicting findings, with some research supporting a positive association between EBV/HPV coinfection and an increased risk of BC [28,30,31], while others have found limited or no evidence to support a significant role for these viral coinfections in BC pathogenesis [19,25]. Accordingly, this study aimed to provide a comprehensive assessment of the prevalence and strength of the association between EBV/HPV coinfection and BC, addressing a critical gap in the literature and highlighting areas for future investigation.

Our meta-analysis revealed an overall prevalence of 14% (95% CI: 12–16%; I^2^ = 90.6%, *p* < 0.0001) for EBV/HPV coinfection in BC patients, based on studies from 13 different countries spanning diverse regions, including the Middle East, East and South Asia, South America, North Africa, as well as contributions from Australia and Croatia. This geographic diversity strengthens the global relevance of our findings. Moreover, to increase the accuracy of our prevalence estimates, we included only studies employing validated methods, such as PCR, as the gold standard to ensure high specificity in detecting EBV and HPV in appropriate tissue samples. Despite this rigorous selection, considerable heterogeneity was observed, likely attributable to differences in detection methods (Figure S2A–C), sampling, and patient demographics. This variability limits the precision of the pooled estimate, highlighting the necessity for larger investigations that control for confounding variables while employing consistent detection methodologies and well-characterized patient cohorts. Future research is essential to produce more robust and generalizable prevalence estimates and to clarify the potential oncogenic role of EBV/HPV coinfection. Nonetheless, our analysis suggests that the presence of both HPV and EBV within breast tissue may not be merely coincidental but could play an active role in breast carcinogenesis.

Our subgroup analysis indicates that the prevalence of EBV/HPV coinfection in breast cancer tissues varied across studies conducted in different regions of the world, reflecting geographical, methodological, and population-based differences. For example, studies from regions with higher EBV prevalence, such as parts of Asia and Africa [34], reported elevated rates of coinfection compared to those from South America. These variations may be attributed to differences in genetic predispositions and may reflect differences in screening practices, socioeconomic factors, and viral exposure risks. In addition, there was an under-representation of studies from regions such as Europe and Australia, and no studies from North America were identified, urging for further research in these areas. Additionally, a temporal trend toward increasing coinfection prevalence was noted, potentially driven by improved diagnostic technologies, changes in sexual behaviors, increased population mobility, and exposure to environmental factors such as pollutants that can increase susceptibility to EBV/HPV coinfection. These findings underscore the need for region-specific public health interventions and longitudinal studies to monitor coinfection trends and their implications for disease burden.

Furthermore, results from our second meta-analysis showed that coinfection with EBV and HPV significantly increased the risk of breast cancer, with a pooled odds ratio indicating a higher likelihood of coinfection in BC cases compared to controls (OR: 5.87; 95% CI: 2.31–14.93). This finding aligns with the oncogenic potential of both EBV and HPV that are known to interfere with cellular processes such as DNA repair, cell cycle regulation, and apoptosis [35,36]. HPV, for instance, expresses E6 and E7 oncoproteins that inactivate tumor suppressor genes like p53 and pRb [37,38], while EBV-encoded proteins such as LMP1 and EBNA1 promote cell proliferation and immune evasion [39,40]. The synergistic effects of these viruses in coinfected tissues may amplify their oncogenic impact, potentially contributing to the initiation or progression of BC. However, the exact mechanisms underlying this synergy remain poorly understood and warrant further investigation through experimental studies.

Findings of this study hold significant implications for clinical and public health strategies. The observed association between EBV/HPV coinfection and BC suggests that viral screening could be explored as a potential biomarker for identifying at-risk populations, especially in regions with high viral prevalence. While HPV vaccination has successfully reduced the incidence of cervical cancer [41], its role in breast cancer prevention remains speculative and requires rigorous evaluation through clinical trials. Additionally, public health initiatives to reduce viral transmission by promoting safe sexual practices and improving hygiene, could indirectly lower the burden of virus-associated BC.

Future research should aim to clarify the biological mechanisms underlying EBV and HPV involvement in BC, including how these viruses interact within breast tissue and whether their combined presence increases oncogenic potential. Investigating host-related factors such as genetic predisposition, immune regulation, and environmental exposures is also critical as they may influence the likelihood of infection, duration of viral activity, and the overall impact on cancer development. Nevertheless, findings of this meta-analysis should be interpreted with caution given the predominance of cross-sectional and case–control studies, which preclude causal inference. The observed EBV/HPV co-infection in breast cancer tissues may therefore represent a secondary or opportunistic event occurring during tumor progression rather than a direct etiological factor. Future longitudinal and mechanistic studies are warranted to clarify whether viral co-infection contributes causally to breast carcinogenesis by assessing the temporal relationship between viral coinfection and disease onset. Additionally, standardizing detection techniques and reporting tumor subtyping would improve study comparability and clinical relevance. Moreover, incorporating viral screening into diagnostic protocols, particularly in high-risk populations, could offer insights into prognosis, help therapeutic decision-making, and support the development of targeted therapies and immunotherapeutic interventions.

## 5. Conclusions

This systematic review and meta-analysis provides important insights into the prevalence and potential role of EBV/HPV coinfection in the development of BC. The prevalence of EBV/HPV coinfection varied widely across studies, with regional differences and an increasing trend over time. Our analysis also revealed a strong association between EBV/HPV coinfection and an elevated risk of BC, with individuals coinfected by both viruses having nearly six times the odds of developing breast cancer compared to those without coinfection. These findings underscore the potential oncogenic role of EBV/HPV coinfection in BC pathogenesis and emphasize the need for further research to elucidate the mechanisms underlying this association. The identification of such associations could pave the way for novel preventive and therapeutic strategies in BC.

## Data Availability

Data is contained within the article or Supplementary Material.

## Supplementary Materials

The following supporting information can be downloaded at: https://www.mdpi.com/article/10.3390/v17121592/s1. Figure S1: Sensitivity analysis of EBV/HPV coinfection prevalence excluding low-quality studies. Figure S2: Sensitivity analysis of EBV/HPV coinfection prevalence using different EBV detection methods. A. Prevalence obtained from PCR-only studies; B. Prevalence obtained from combined methods; C. Prevalence obtained from alternative methods. Figure S3: Sensitivity analysis of EBV/HPV coinfection prevalence using different viral gene targets. A. Prevalence obtained from PCR targeting the E6/E7 region; B. Prevalence obtained from PCR targeting the L1 region. Figure S4: Funnel plot of publication bias for EBV/HPV co-infection in breast cancer. Figure S5: Forest plot of the prevalence of human papillomavirus and Epstein–Barr virus coinfection among patients with breast cancer by weighting results according to study quality. Table S1: PRISMA Checklist. Table S2: Excluded articles at full text screening. Table S3: Characteristics of included studies and population profiles. Table S4: Characteristics of included samples. Table S5: Quality assessment of included articles using Joanna Briggs Institute’s checklists. A. Quality assessment for case–control studies. JBI’s criteria for case–control studies: (i) Were the groups comparable other than the presence of disease in cases or the absence of disease in controls?; (ii) Were cases and controls matched appropriately?; (iii) Were the same criteria used for identification of cases and controls?; (iv) Was exposure measured in a standard, valid and reliable way?; (v) Was exposure measured in the same way for cases and controls?; (vi) Were confounding factors identified?; (vii) Were strategies to deal with confounding factors stated?; (viii) Were outcomes assessed in a standard, valid and reliable way for cases and controls?; (ix) Was the exposure period of interest long enough to be meaningful?; (x) Was appropriate statistical analysis used?; Score, n (%) B. Quality assessment for analytical cross-sectional studies. JBI’s criteria for cross-sectional studies: (i) Were the criteria for inclusion in the sample clearly defined?; (ii) Were the study subjects and the setting described in detail?; (iii) Was the exposure measured in a valid and reliable way?; (iv) Were objective, standard criteria used for measurement of the condition?; (v) Were confounding factors identified?; (vi) Were strategies to deal with confounding factors stated?; (vii) Were the outcomes measured in a valid and reliable way?; (viii) Was appropriate statistical analysis used? Score, n (%); Y, Yes; N, No; UC, Unclear; NA, Not Applicable. Table S6: Summary of methodological approaches reported for HPV and EBV detection in BC patients. Reference [42] is cited in the supplementary materials.

## Abbreviations

The following abbreviations are used in this manuscript:

BC	Breast cancer
EBV	Epstein–Barr virus
HPV	human papillomavirus
PCR	polymerase chain reaction

## Appendix A

Search strategy

**PubMed**
(“Breast Neoplasms” [Mesh] OR Breast Neoplasm OR Breast Tumors OR Breast Tumor OR Breast Neoplasms OR Breast Cancer OR Mammary Cancer OR Mammary Cancers OR Breast Malignant Neoplasm OR Breast Malignant Neoplasms OR Breast Malignant Tumor OR Breast Malignant Tumors OR Human Mammary Carcinomas OR Human Mammary Carcinoma OR Human Mammary Neoplasm OR Human Mammary Neoplasms OR Breast Carcinoma OR Breast Carcinomas) AND (“Herpesvirus 4, Human” [Mesh] OR Epstein-Barr Virus OR Epstein Barr Virus OR Burkitt Lymphoma Virus OR Burkitt’s Lymphoma Virus OR Burkitts Lymphoma Virus OR E-B Virus OR Infectious Mononucleosis Virus OR Infectious Mononucleosis Viruses OR HHV-4 OR HHV4 OR Human Herpesvirus 4 OR Burkitt Herpesvirus OR EBV) AND (“Human Papillomavirus Viruses” [Mesh] OR Human Papillomavirus OR HPV Human Papillomavirus OR Human Papilloma Virus or HPV).

**Web of Science**
(“Breast Neoplasm” OR “Breast Tumors” OR “Breast Tumor” OR “Breast Neoplasms” OR “Breast Cancer” OR “Mammary Cancer” OR “Mammary Cancers” OR “Breast Malignant Neoplasm” OR “Breast Malignant Neoplasms” OR “Breast Malignant Tumor” OR “Breast Malignant Tumors” OR “Human Mammary Carcinomas” OR “Human Mammary Carcinoma” OR “Human Mammary Neoplasm” OR “Human Mammary Neoplasms” OR “Breast Carcinoma” OR “Breast Carcinomas”) (All Fields) AND (“Epstein-Barr Virus” OR “Epstein Barr Virus” OR “Burkitt Lymphoma Virus” OR “Burkitt’s Lymphoma Virus” OR “Burkitts Lymphoma Virus” OR “E-B Virus” OR “Infectious Mononucleosis Virus” OR “Infectious Mononucleosis Viruses” OR “HHV-4” OR “HHV4” OR “Human Herpesvirus 4” OR “Burkitt Herpesvirus” OR “EBV”) (All Fields) AND (“Human Papillomavirus” OR “HPV Human Papillomavirus” OR “Human Papilloma Virus” or “HPV”) (All Fields) AND LANGUAGE: (English)
**Scopus**
TITLE-ABS-KEY (“Breast Neoplasm” OR “Breast Tumors” OR “Breast Tumor” OR “Breast Neoplasms” OR “Breast Cancer” OR “Mammary Cancer” OR “Mammary Cancers” OR “Breast Malignant Neoplasm” OR “Breast Malignant Neoplasms” OR “Breast Malignant Tumor” OR “Breast Malignant Tumors” OR “Human Mammary Carcinomas” OR “Human Mammary Carcinoma” OR “Human Mammary Neoplasm” OR “Human Mammary Neoplasms” OR “Breast Carcinoma” OR “Breast Carcinomas”) AND TITLE-ABS-KEY (“Epstein-Barr Virus” OR “Epstein Barr Virus” OR “Burkitt Lymphoma Virus” OR “Burkitt’s Lymphoma Virus” OR “Burkitts Lymphoma Virus” OR “E-B Virus” OR “Infectious Mononucleosis Virus” OR “Infectious Mononucleosis Viruses” OR “HHV-4” OR “HHV4” OR “Human Herpesvirus 4” OR “Burkitt Herpesvirus” OR “EBV”) AND TITLE-ABS-KEY (“Human Papillomavirus” OR “HPV Human Papillomavirus” OR “Human Papilloma Virus” or “HPV”) AND LANGUAGE: (English)
**Embase**
(‘Breast Neoplasm’ OR ‘Breast Tumors’ OR ‘Breast Tumor’ OR ‘Breast Neoplasms’ OR ‘Breast Cancer’ OR ‘Mammary Cancer’ OR ‘Mammary Cancers’ OR ‘Breast Malignant Neoplasm’ OR ‘Breast Malignant Neoplasms’ OR ‘Breast Malignant Tumor’ OR ‘Breast Malignant Tumors’ OR ‘Human Mammary Carcinomas’ OR ‘Human Mammary Carcinoma’ OR ‘Human Mammary Neoplasm’ OR ‘Human Mammary Neoplasms’ OR ‘Breast Carcinoma’ OR ‘Breast Carcinomas’) AND (‘Epstein-Barr Virus’ OR ‘Epstein Barr Virus’ OR ‘Burkitt Lymphoma Virus’ OR ‘Burkitts Lymphoma Virus’ OR ‘E-B Virus’ OR ‘Infectious Mononucleosis Virus’ OR ‘Infectious Mononucleosis Viruses’ OR ‘HHV-4′ OR ‘HHV4′ OR ‘Human Herpesvirus 4′ OR ‘Burkitt Herpesvirus’ OR ‘EBV’) AND (‘Human Papillomavirus’ OR ‘HPV Human Papillomavirus’ OR ‘Human Papilloma Virus’ or ‘HPV’)

## References

[1] Filho A.M., Laversanne M., Ferlay J., Colombet M., Pineros M., Znaor A., Parkin D.M., Soerjomataram I., Bray F. (2025). The GLOBOCAN 2022 cancer estimates: Data sources, methods, and a snapshot of the cancer burden worldwide. Int. J. Cancer.

[2] Obeagu E.I., Obeagu G.U. (2024). Breast cancer: A review of risk factors and diagnosis. Medicine.

[3] Moore P.S., Chang Y. (2010). Why do viruses cause cancer? Highlights of the first century of human tumour virology. Nat. Rev. Cancer.

[4] Morales-Sanchez A., Fuentes-Panana E.M. (2014). Human viruses and cancer. Viruses.

[5] Blanco R., Carrillo-Beltran D., Corvalan A.H., Aguayo F. (2021). High-Risk Human Papillomavirus and Epstein-Barr Virus Coinfection: A Potential Role in Head and Neck Carcinogenesis. Biology.

[6] Munz C. (2023). Modulation of Epstein-Barr-Virus (EBV)-Associated Cancers by Co-Infections. Cancers.

[7] Cyprian F.S., Al-Farsi H.F., Vranic S., Akhtar S., Al Moustafa A.E. (2018). Epstein-Barr Virus and Human Papillomaviruses Interactions and Their Roles in the Initiation of Epithelial-Mesenchymal Transition and Cancer Progression. Front. Oncol..

[8] Yeo-Teh N.S.L., Ito Y., Jha S. (2018). High-Risk Human Papillomaviral Oncogenes E6 and E7 Target Key Cellular Pathways to Achieve Oncogenesis. Int. J. Mol. Sci..

[9] Luo Y., Liu Y., Wang C., Gan R. (2021). Signaling pathways of EBV-induced oncogenesis. Cancer Cell Int..

[10] Shair K.H., Bendt K.M., Edwards R.H., Bedford E.C., Nielsen J.N., Raab-Traub N. (2007). EBV latent membrane protein 1 activates Akt, NFkappaB, and Stat3 in B cell lymphomas. PLoS Pathog..

[11] Apcher S., Daskalogianni C., Manoury B., Fahraeus R. (2010). Epstein Barr virus-encoded EBNA1 interference with MHC class I antigen presentation reveals a close correlation between mRNA translation initiation and antigen presentation. PLoS Pathog..

[12] Jung Y.S., Kato I., Kim H.R. (2013). A novel function of HPV16-E6/E7 in epithelial-mesenchymal transition. Biochem. Biophys. Res. Commun..

[13] Horikawa T., Yoshizaki T., Kondo S., Furukawa M., Kaizaki Y., Pagano J.S. (2011). Epstein-Barr Virus latent membrane protein 1 induces Snail and epithelial-mesenchymal transition in metastatic nasopharyngeal carcinoma. Br. J. Cancer.

[14] Zheng M., Huang J., Tong A., Yang H. (2019). Oncolytic Viruses for Cancer Therapy: Barriers and Recent Advances. Mol. Ther. Oncolytics.

[15] Newcombe R.G. (1998). Two-sided confidence intervals for the single proportion: Comparison of seven methods. Stat. Med..

[16] Aguayo F., Khan N., Koriyama C., Gonzalez C., Ampuero S., Padilla O., Solis L., Eizuru Y., Corvalan A., Akiba S. (2011). Human papillomavirus and Epstein-Barr virus infections in breast cancer from chile. Infect. Agent. Cancer.

[17] Corbex M., Bouzbid S., Traverse-Glehen A., Aouras H., McKay-Chopin S., Carreira C., Lankar A., Tommasino M., Gheit T. (2014). Prevalence of papillomaviruses, polyomaviruses, and herpesviruses in triple-negative and inflammatory breast tumors from algeria compared with other types of breast cancer tumors. PLoS ONE.

[18] Peng J., Wang T., Zhu H., Guo J., Li K., Yao Q., Lv Y., Zhang J., He C., Chen J. (2014). Multiplex PCR/mass spectrometry screening of biological carcinogenic agents in human mammary tumors. J. Clin. Virol..

[19] Fimereli D., Gacquer D., Fumagalli D., Salgado R., Rothe F., Larsimont D., Sotiriou C., Detours V. (2015). No significant viral transcription detected in whole breast cancer transcriptomes. BMC Cancer.

[20] Shet T., Pai T., Shetty O., Desai S. (2016). Lymphoepithelioma-like carcinoma of breast-evaluation for Epstein-Barr virus-encoded RNA, human papillomavirus, and markers of basal cell differentiation. Ann. Diagn. Pathol..

[21] Ladera M., Fernandes A., López M., Pesci-Feltri A., Ávila M., Correnti M. (2017). Presence of human papillomavirus and Epstein-Barr virus in breast cancer biopsies as potential risk factors. Gac. Mex. De Oncol..

[22] Naushad W., Surriya O., Sadia H. (2017). Prevalence of EBV, HPV and MMTV in Pakistani breast cancer patients: A possible etiological role of viruses in breast cancer. Infect. Genet. Evol..

[23] Metwally S.A., Abo-Shadi M.A., Abdel Fattah N.F., Barakat A.B., Rabee O.A., Osman A.M., Helal A.M., Hashem T., Moneer M.M., Chehadeh W. (2021). Presence of HPV, EBV and HMTV Viruses Among Egyptian Breast Cancer Women: Molecular Detection and Clinical Relevance. Infect. Drug Resist..

[24] Nakhaie M., Makvandi M., Charostad J., Arabzadeh S.A.M., Motamedfar A., Kaydani G.A. (2020). Downregulation of mir-143/145 cluster in breast carcinoma specimens: Putative role of dna oncoviruses. Jundishapur J. Microbiol..

[25] Fani M., Sadooni R., Nejad M.H., Mirzavieh N.N., Mobarak S., Pakzad R., Erfani Y., Salmanzadeh S., Abbasi S. (2023). Two Oncoviruses of HPV and EBV in Breast Cancer: An Iran-based Study. J. Liaquat Univ. Med. Health Sci..

[26] Khasawneh A.I., Himsawi N., Sammour A., Al Shboul S., Alorjani M., Al-Momani H., Shahin U., Al-Momani H., Alotaibi M.R., Saleh T. (2024). Association of Human Papilloma Virus, Cytomegalovirus, and Epstein-Barr Virus with Breast Cancer in Jordanian Women. Medicina.

[27] Gupta I., Jabeen A., Al-Sarraf R., Farghaly H., Vranic S., Sultan A.A., Al Moustafa A.E., Al-Thawadi H. (2021). The co-presence of high-risk human papillomaviruses and Epstein-Barr virus is linked with tumor grade and stage in Qatari women with breast cancer. Hum. Vaccin. Immunother..

[28] Nagi K., Gupta I., Jurdi N., Jabeen A., Yasmeen A., Batist G., Vranic S., Al-Moustafa A.E. (2021). High-risk human papillomaviruses and Epstein-Barr virus in breast cancer in Lebanese women and their association with tumor grade: A molecular and tissue microarray study. Cancer Cell Int..

[29] Lawson J.S., Glenn W.K. (2017). Multiple oncogenic viruses are present in human breast tissues before development of virus associated breast cancer. Infect. Agent. Cancer.

[30] Gupta I., Ulamec M., Peric-Balja M., Ramic S., Al Moustafa A.E., Vranic S., Al-Farsi H.F. (2021). Presence of high-risk HPVs, EBV, and MMTV in human triple-negative breast cancer. Hum. Vaccin. Immunother..

[31] Al Moustafa A.E., Al-Antary N., Aboulkassim T., Akil N., Batist G., Yasmeen A. (2016). Co-prevalence of Epstein-Barr virus and high-risk human papillomaviruses in Syrian women with breast cancer. Hum. Vaccin. Immunother..

[32] Ayee R., Ofori M.E.O., Wright E., Quaye O. (2020). Epstein Barr Virus Associated Lymphomas and Epithelia Cancers in Humans. J. Cancer.

[33] Baba S.K., Alblooshi S.S.E., Yaqoob R., Behl S., Al Saleem M., Rakha E.A., Malik F., Singh M., Macha M.A., Akhtar M.K. (2025). Human papilloma virus (HPV) mediated cancers: An insightful update. J. Transl. Med..

[34] Farahmand M., Monavari S.H., Shoja Z., Ghaffari H., Tavakoli M., Tavakoli A. (2019). Epstein-Barr virus and risk of breast cancer: A systematic review and meta-analysis. Future Oncol..

[35] Hollingworth R., Grand R.J. (2015). Modulation of DNA damage and repair pathways by human tumour viruses. Viruses.

[36] Sitz J., Blanchet S.A., Gameiro S.F., Biquand E., Morgan T.M., Galloy M., Dessapt J., Lavoie E.G., Blondeau A., Smith B.C. (2019). Human papillomavirus E7 oncoprotein targets RNF168 to hijack the host DNA damage response. Proc. Natl. Acad. Sci. USA.

[37] Fischer M., Uxa S., Stanko C., Magin T.M., Engeland K. (2017). Human papilloma virus E7 oncoprotein abrogates the p53-p21-DREAM pathway. Sci. Rep..

[38] Slebos R.J., Lee M.H., Plunkett B.S., Kessis T.D., Williams B.O., Jacks T., Hedrick L., Kastan M.B., Cho K.R. (1994). p53-dependent G1 arrest involves pRB-related proteins and is disrupted by the human papillomavirus 16 E7 oncoprotein. Proc. Natl. Acad. Sci. USA.

[39] Damania B., Kenney S.C., Raab-Traub N. (2022). Epstein-Barr virus: Biology and clinical disease. Cell.

[40] Wang L.W., Jiang S., Gewurz B.E. (2017). Epstein-Barr Virus LMP1-Mediated Oncogenicity. J. Virol..

[41] Lei J., Ploner A., Elfstrom K.M., Wang J., Roth A., Fang F., Sundstrom K., Dillner J., Sparen P. (2020). HPV Vaccination and the Risk of Invasive Cervical Cancer. N. Engl. J. Med..

[42] Page M.J., McKenzie J.E., Bossuyt P.M., Boutron I., Hoffmann T.C., Mulrow C.D., Shamseer L., Tetzlaff J.M., Akl E.A., Brennan S.E. (2021). The PRISMA 2020 statement: An updated guideline for reporting systematic reviews. BMJ.

