# Supplementary material for: Global Prevalence and Cancer Risk of Epstein–Barr Virus and Human Papillomavirus Coinfection in Breast Cancer: A Systematic Review and Meta-Analysis
Source: Viruses. 2025 Dec 8;17(12):1592. doi: 10.3390/v17121592 (PMC12737756; doi:10.3390/v17121592)
Supplement: Supplementary file 1 [file viruses-17-01592-s001.zip › viruses-3936848-supplementary.pdf]

Supplementary Materials for

Global Prevalence and Cancer Risk of Epstein-Barr virus and Human papillomavirus Coinfection in Breast Cancer: A Systematic Review and Meta-analysis

Abdelrahman A. Karen[1#], Albara S. Elkhalaf[1#], Omar Tluli[1], Omar Sorour[1], Abdulnaser Fakhrou[2], Mohammed Imad Malki[1] and Karim Nagi[1*]

[1] Department of Basic Medical Sciences, College of Medicine, QU Health, Qatar University, Doha, Qatar.

[2] Psychological Sciences, College of Education, Qatar University, Doha, Qatar

[#] Equal contribution

*Corresponding author. Email: knagi@qu.edu.qa

Supplementary Materials contains:

- 5 Supplementary Figures.
- 6 Supplementary Tables.

Figure S1: Sensitivity analysis of EBV/HPV coinfection prevalence excluding low-quality studies.

Figure S2: Sensitivity analysis of EBV/HPV coinfection prevalence using different EBV detection methods. A. Prevalence obtained from PCR-only studies; B. Prevalence obtained from combined methods; C. Prevalence obtained from alternative methods.

Figure S3: Sensitivity analysis of EBV/HPV coinfection prevalence using different viral gene targets. A. Prevalence obtained from PCR targeting the E6/E7 region; B. Prevalence obtained from PCR targeting the L1 region.

Figure S4: Funnel plot of publication bias for EBV/HPV co-infection in breast cancer.

Figure S5: Forest plot of the prevalence of human papillomavirus and Epstein-Barr virus coinfection among patients with breast cancer by weighting results according to study quality.

Table S1: PRISMA Checklist.

Table S2: Excluded articles at full text screening.

Table S3: Characteristics of included studies and population profiles.

Table S4: Characteristics of included samples.

Table S5: Quality assessment of included articles using Joanna Briggs Institute's checklists. A. Quality assessment for case-control studies. JBI's criteria for case-control studies: i) Were the groups comparable other than the presence of disease in cases or the absence of disease in controls?; ii) Were cases and controls matched appropriately?; iii) Were the same criteria used for identification of cases and controls?; iv) Was exposure measured in a standard, valid and reliable way?; v) Was exposure measured in the same way for cases and controls?; vi) Were confounding factors identified?; vii) Were strategies to deal with confounding factors stated?; viii) Were outcomes assessed in a standard, valid and reliable way for cases and controls?; ix) Was the exposure period of interest long enough to be meaningful?; x) Was appropriate statistical analysis used?; Score, n (%) B. Quality assessment for analytical cross-sectional

studies. JBI's criteria for cross-sectional studies: i) Were the criteria for inclusion in the sample clearly defined?; ii) Were the study subjects and the setting described in detail?; iii) Was the exposure measured in a valid and reliable way?; iv) Were objective, standard criteria used for measurement of the condition?; v) Were confounding factors identified?; vi) Were strategies to deal with confounding factors stated?; vii) Were the outcomes measured in a valid and reliable way?; viii) Was appropriate statistical analysis used? Score, n (%)

Y, Yes; N, No; UC, Unclear; NA, Not Applicable.

Table S6: Summary of methodological approaches reported for HPV and EBV detection in BC patients.

Figure S1

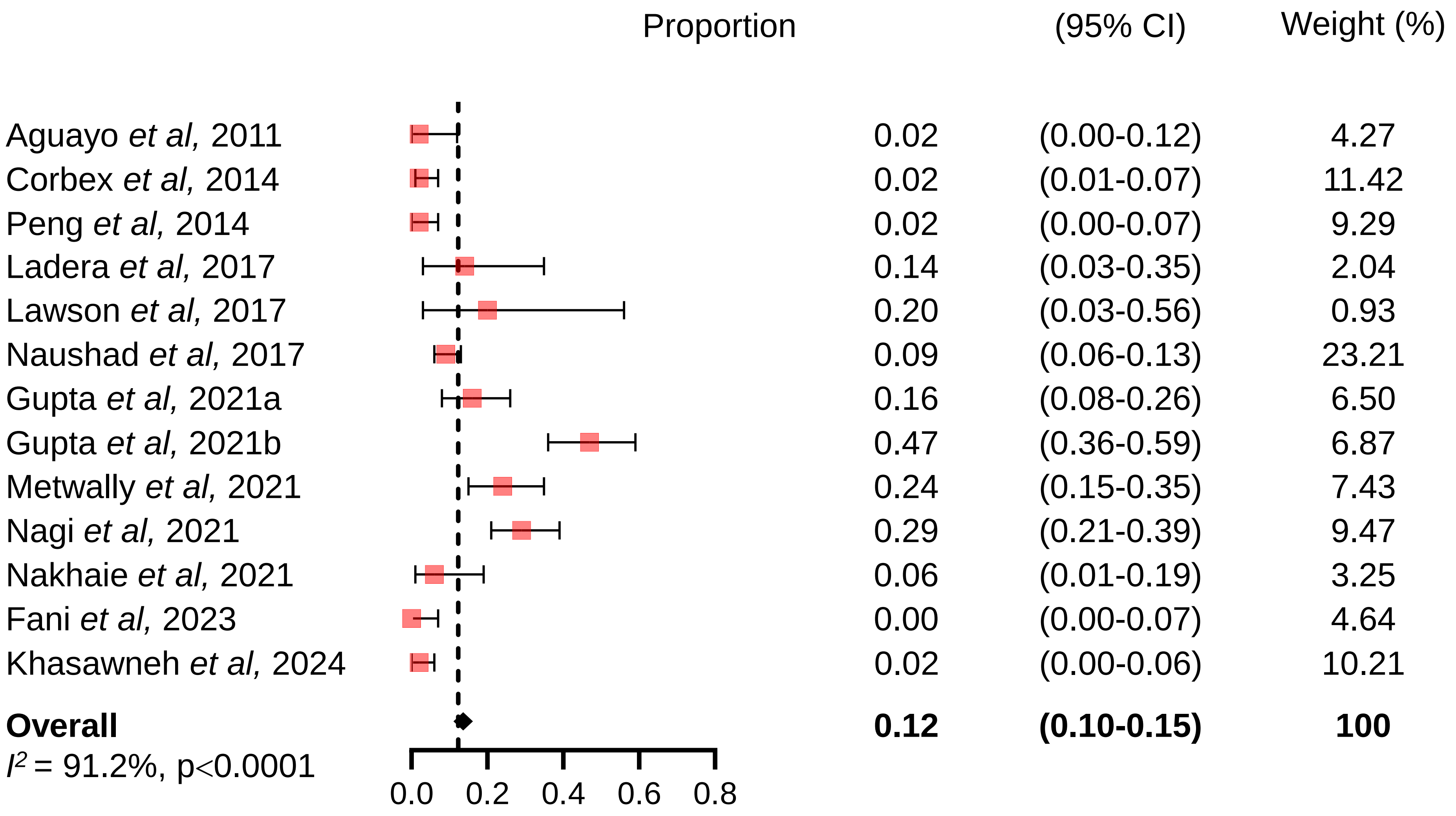

Figure S1. Sensitivity analysis of EBV/HPV coinfection prevalence excluding low-quality studies.

Figure S2

A.

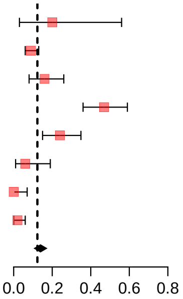

	Proportion	(95% CI)	Weight (%)
Lawson *et al,* 2017	0.20	(0.03-0.56)	1.47
Naushad *et al,* 2017	0.09	(0.06-0.13)	36.82
Gupta *et al,* 2021a	0.16	(0.08-0.26)	10.31
Gupta *et al,* 2021b	0.47	(0.36-0.59)	10.90
Metwally *et al,* 2021	0.24	(0.15-0.35)	11.78
Nakhaie *et al,* 2021	0.06	(0.01-0.19)	5.15
Fani *et al,* 2023	0.00	(0.00-0.07)	7.36
Khasawneh *et al,* 2024	0.02	(0.00-0.06)	16.20
**Overall**	**0.138**	**(0.11-0.17)**	**100**
I^2 = 91.6%, p<0.0001			

B.

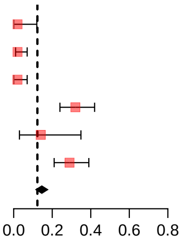

	Proportion	(95% CI)	Weight (%)
Aguayo *et al,* 2011	0.02	(0.00-0.12)	9.18
Corbex *et al,* 2014	0.02	(0.01-0.07)	24.55
Peng *et al,* 2014	0.02	(0.00-0.07)	19.96
Al Moustafa *et al,* 2016	0.32	(0.24-0.42)	21.56
Ladera *et al,* 2017	0.14	(0.03-0.35)	4.39
Nagi *et al,* 2021	0.29	(0.21-0.39)	20.36
**Overall**	**0.148**	**(0.12-0.18)**	**100**
I^2 = 91.1%, p<0.0001			

C.

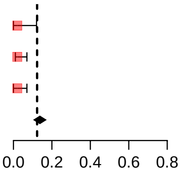

	Proportion	(95% CI)	Weight (%)
Fimereli *et al,* 2015	0.00	(0.00-0.07)	9.60
Shet *et al,* 2016	0.00	(0.00-0.52)	25.68
Olivera *et al,* 2022	0.00	(0.00-0.07)	20.88
**Overall**	**0.000**	**(0.00-0.03)**	**100**
I^2 = 0%, p=1.0			

Figure S2. Sensitivity analysis of EBV/HPV coinfection prevalence using different EBV detection methods. A. Prevalence obtained from PCR-only studies; B. Prevalence obtained from combined methods; C. Prevalence obtained from alternative methods.

Figure S3

A.

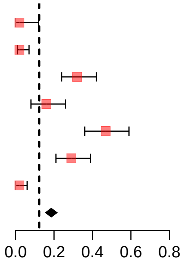

Study	Proportion	(95% CI)	Weight (%)
Aguayo *et al,* 2011	0.02	(0.00-0.12)	7.27
Corbex *et al,* 2014	0.02	(0.01-0.07)	19.43
Al Moustafa *et al,* 2016	0.32	(0.24-0.42)	17.06
Gupta *et al,* 2021a	0.16	(0.08-0.26)	11.06
Gupta *et al,* 2021b	0.47	(0.36-0.59)	11.69
Nagi *et al,* 2021	0.29	(0.21-0.39)	16.11
Khasawneh *et al,* 2024	0.02	(0.00-0.06)	17.38
**Overall**	**0.18**	**(0.16-0.22)**	**100**

I^2 = 95.2%, p<0.001

B.

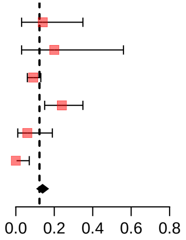

Study	Proportion	(95% CI)	Weight (%)
Ladera *et al,* 2017	0.14	(0.03-0.35)	4.92
Lawson *et al,* 2017	0.20	(0.03-0.56)	2.24
Naushad *et al,* 2017	0.09	(0.06-0.13)	55.93
Metwally *et al,* 2021	0.24	(0.15-0.35)	17.90
Nakhaie *et al,* 2021	0.06	(0.01-0.19)	7.83
Fani *et al,* 2023	0.00	(0.00-0.07)	11.19
**Overall**	**0.11**	**(0.08-0.14)**	**100**

I^2 = 82.5%, p<0.0001

Figure S3. Sensitivity analysis of EBV/HPV coinfection prevalence using different viral gene targets.
A. Prevalence obtained from PCR targeting the E6/E7 region; B. Prevalence obtained from PCR targeting the L1 region.

Figure S4

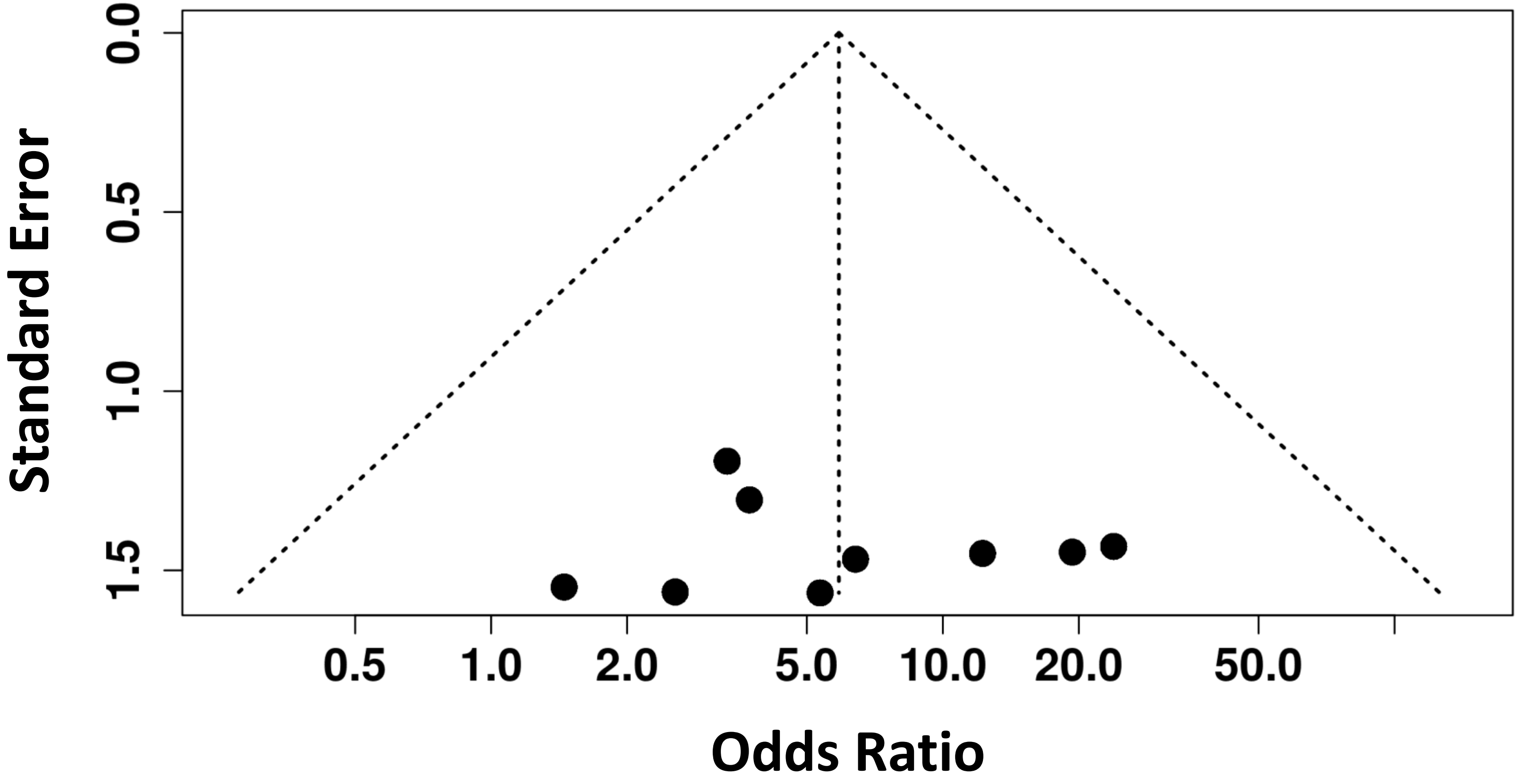

Figure S4. Funnel plot of publication bias for EBV/HPV co-infection in breast cancer.

Figure S5

	Quality Score	Odds Ratio	(95% CI)	Weight
Peng *et al*, 2014	70	2.56	(0.12-54.41)	11.2%
Ladera *et al*, 2017	80	3.32	(0.32-34.65)	12.8%
Lawson *et al*, 2017	67	3.75	(0.29-47.99)	10.6%
Naushad *et al*, 2017	70	23.86	(1.44-396.37)	11.2%
Gupta *et al*, 2021a	60	6.38	(0.36-114.02)	9.6%
Metwally *et al*, 2021	70	19.34	(1.13-331.20)	11.2%
Nagi *et al*, 2021	70	12.20	(0.71-211.07)	11.2%
Nakhaie *et al*, 2021	70	5.30	(0.25-114.47)	11.2%
Khasawneh *et al*, 2024	70	1.41	(0.07-30.06)	11.2%
**Overall**		**5.86**	**(2.31-14.88)**	**100%**

Heterogeneity: $Tau^2 = 0.00$; $Chi^2 = 3.37$, df=8 (p=0.91); $I^2 = 0\%$
Test for overall effect: Z= 3.73 ($p < 0.001$)

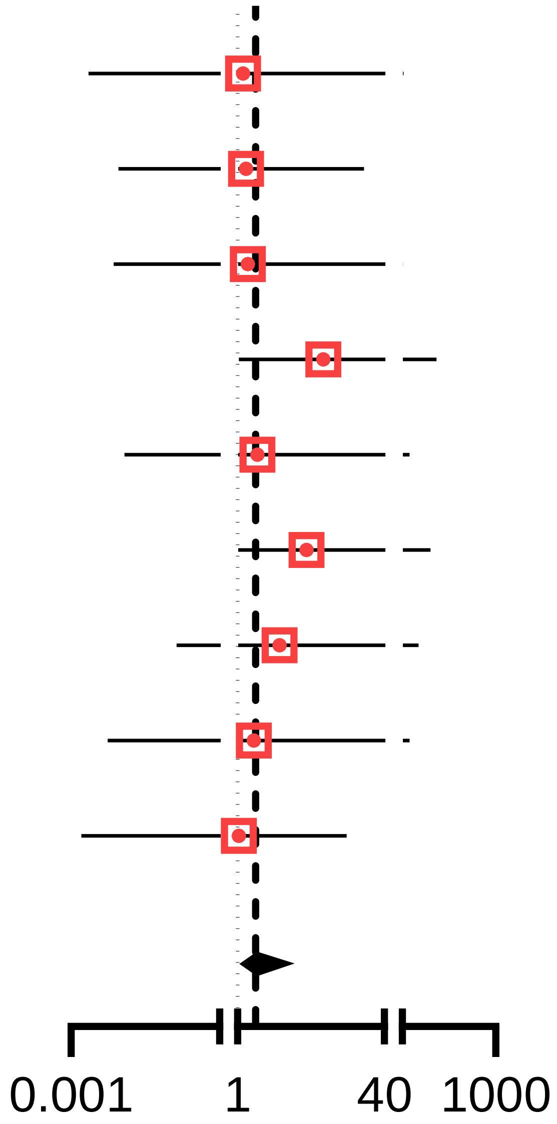

Figure S5: Forest plot of the prevalence of human papillomavirus and Epstein-Barr virus coinfection among patients with breast cancer by weighting results according to study quality.

Table S1. PRISMA checklist

	Item #	Checklist item	Location where item is reported
**TITLE**			
Title	1	Identify the report as a systematic review.	1
**ABSTRACT**			
Abstract	2	See the PRISMA 2020 for Abstracts checklist.	1
**INTRODUCTION**			
Rationale	3	Describe the rationale for the review in the context of existing knowledge.	1
Objectives	4	Provide an explicit statement of the objective(s) or question(s) the review addresses.	2
**METHODS**			
Eligibility criteria	5	Specify the inclusion and exclusion criteria for the review and how studies were grouped for the syntheses.	3
Information sources	6	Specify all databases, registers, websites, organisations, reference lists and other sources searched or consulted to identify studies. Specify the date when each source was last searched or consulted.	3
Search strategy	7	Present the full search strategies for all databases, registers and websites, including any filters and limits used.	Appendix A
Selection process	8	Specify the methods used to decide whether a study met the inclusion criteria of the review, including how many reviewers screened each record and each report retrieved, whether they worked independently, and if applicable, details of automation tools used in the process.	3-4
Data collection process	9	Specify the methods used to collect data from reports, including how many reviewers collected data from each report, whether they worked independently, any processes for obtaining or confirming data from study investigators, and if applicable, details of automation tools used in the process.	4
Data items	10a	List and define all outcomes for which data were sought. Specify whether all results that were compatible with each outcome domain in each study were sought (e.g. for all measures, time points, analyses), and if not, the methods used to decide which results to collect.	4-5
	10b	List and define all other variables for which data were sought (e.g. participant and intervention characteristics, funding sources). Describe any assumptions made about any missing or unclear information.	4-5 and Table S3
Study risk of bias assessment	11	Specify the methods used to assess risk of bias in the included studies, including details of the tool(s) used, how many reviewers assessed each study and whether they worked independently, and if applicable, details of automation tools used in the process.	4-5 and Table S5
Effect measures	12	Specify for each outcome the effect measure(s) (e.g. risk ratio, mean difference) used in the synthesis or presentation of results.	4-5
Synthesis methods	13a	Describe the processes used to decide which studies were eligible for each synthesis (e.g. tabulating the study intervention characteristics and comparing against the planned groups for each synthesis (item #5)).	4-5
	13b	Describe any methods required to prepare the data for presentation or synthesis, such as handling of missing summary statistics, or data conversions.	4-5
	13c	Describe any methods used to tabulate or visually display results of individual studies and syntheses.	4-5
	13d	Describe any methods used to synthesize results and provide a rationale for the choice(s). If meta-analysis was performed, describe the model(s), method(s) to identify the presence and extent of statistical heterogeneity, and software package(s) used.	4-5
	13e	Describe any methods used to explore possible causes of heterogeneity among study results (e.g. subgroup analysis, meta-regression).	5
	13f	Describe any sensitivity analyses conducted to assess robustness of the synthesized results.	5
Reporting bias assessment	14	Describe any methods used to assess risk of bias due to missing results in a synthesis (arising from reporting biases).	5-6
Certainty assessment	15	Describe any methods used to assess certainty (or confidence) in the body of evidence for an outcome.	5

	Item #	Checklist item	Location where item is reported
**RESULTS**			
Study selection	16a	Describe the results of the search and selection process, from the number of records identified in the search to the number of studies included in the review, ideally using a flow diagram.	5 and Figure 1
	16b	Cite studies that might appear to meet the inclusion criteria, but which were excluded, and explain why they were excluded.	5 and Table S2
Study characteristics	17	Cite each included study and present its characteristics.	5 and Table S3
Risk of bias in studies	18	Present assessments of risk of bias for each included study.	5-6
Results of individual studies	19	For all outcomes, present, for each study: (a) summary statistics for each group (where appropriate) and (b) an effect estimate and its precision (e.g. confidence/credible interval), ideally using structured tables or plots.	6-9 and Figures 2-5
Results of syntheses	20a	For each synthesis, briefly summarise the characteristics and risk of bias among contributing studies.	9 and Table S5
	20b	Present results of all statistical syntheses conducted. If meta-analysis was done, present for each the summary estimate and its precision (e.g. confidence/credible interval) and measures of statistical heterogeneity. If comparing groups, describe the direction of the effect.	5-9 and Figures 2-5
	20c	Present results of all investigations of possible causes of heterogeneity among study results.	5-7,-9 and Figures S2-S3
	20d	Present results of all sensitivity analyses conducted to assess the robustness of the synthesized results.	5-7, 9 and Fig S1-S5
Reporting biases	21	Present assessments of risk of bias due to missing results (arising from reporting biases) for each synthesis assessed.	5-6
Certainty of evidence	22	Present assessments of certainty (or confidence) in the body of evidence for each outcome assessed.	5-7, 9
**DISCUSSION**			
Discussion	23a	Provide a general interpretation of the results in the context of other evidence.	9, 12
	23b	Discuss any limitations of the evidence included in the review.	10
	23c	Discuss any limitations of the review processes used.	10
	23d	Discuss implications of the results for practice, policy, and future research.	11-12
**OTHER INFORMATION**			
Registration and protocol	24a	Provide registration information for the review, including register name and registration number, or state that the review was not registered.	3
	24b	Indicate where the review protocol can be accessed, or state that a protocol was not prepared.	3
	24c	Describe and explain any amendments to information provided at registration or in the protocol.	3
Support	25	Describe sources of financial or non-financial support for the review, and the role of the funders or sponsors in the review.	13
Competing interests	26	Declare any competing interests of review authors.	13
Availability of data, code and other materials	27	Report which of the following are publicly available and where they can be found: template data collection forms; data extracted from included studies; data used for all analyses; analytic code; any other materials used in the review.	12

From: Page MJ, McKenzie JE, Bossuyt PM, Boutron I, Hoffmann TC, Mulrow CD, et al. The PRISMA 2020 statement: an updated guideline for reporting systematic reviews. BMJ 2021;372:n71. doi: 10.1136/bmj.n71. This work is licensed under CC BY 4.0. To view a copy of this license, visit https://creativecommons.org/licenses/by/4.0/

Table S2: Excluded articles at full text screening.

Study DOI	Title	Reason for exclusion
10.4269/ajtmh.21-0692	Cancer Screening in Refugees and Immigrants: A Global Perspective	Screening in non-BC tissues
10.1038/modpathol.2011.135	Lymphadenoma of the salivary gland: Clinicopathological and immunohistochemical analysis of 33 tumors	Screening in non-BC tissues
10.1016/j.canlet.2024.217318	Precision meets repurposing: Innovative approaches in human papillomavirus and Epstein-Barr virus-driven cancer therapy	Screening in non-BC tissues
10.1089/vim.2019.0156	Antiviral T Cell Receptor Complementarity Determining Region-3 Sequences Are Associated with a Worse Cancer Outcome: A Pancancer Analysis	Screening in non-BC tissues
10.1080/1744666X.2018.1519394	Managing cancer risk in patients with systemic lupus erythematous	Screening in non-BC tissues
10.1097/PGP.0000000000000446	Inflammatory Stroma of Lymphoepithelioma-like Carcinoma of the Cervix: Immunohistochemical Study of 3 Cases and Review of the Literature	Screening in non-BC tissues
10.2174/157016205774370384	AIDS related viruses, their association with leukemia, and raf signaling	Screening in non-BC tissues
10.1016/j.humpath.2007.08.006	Lymphoepithelioma-like carcinoma of the breast: not Epstein-Barr virus–, but human papilloma virus–positive	Less than 5 BC cases
10.1016/j.ejphar.2022.175393	Oncolytic viruses as emerging therapy against cancers including Oncovirus-induced cancers	Usage of cell/animal models
10.1016/j.gine.2019.06.001	Breast cancer and virus, yes but no; [Cáncer de mama y virus, sí pero no]	Language other than English
10.1186/s12885-015-1176-2	No significant viral transcription detected in whole breast cancer transcriptomes	Non-PCR viral detection
10.1016/j.anndiagpath.2016.05.006	Lymphoepithelioma-like carcinoma of breast-evaluation for Epstein-Barr virus-encoded RNA, human papillomavirus, and markers of basal cell differentiation	Non-PCR viral detection
10.31557/APJCP.2022.23.7.2351	High Frequency of Epstein-Barr Virus and Absence of Papillomavirus in Breast Cancer Patients from Brazilian Northeast	Non-PCR viral detection

Table S3. Characteristics of included studies and population profiles

First author, year	Country of origin	Clinical profile of BC patients	Mean BC patients age	Type of study
Aguayo *et al,* 2011	Chile	Patients with ductal, lobulillar or mucinous BC	58.70	Cross-sectional
Corbex *et al,* 2014	Algeria	Patients with Inflammatory & triple-negative BC (2008-2011)	48.80	Cross-sectional
Peng *et al,* 2014	China	Patients with BC treated surgically (October 2010- October 2011)	Not specified	Case–control
Al Moustafa *et al,* 2016	Syria	Patients with invasive and in situ BC	Not specified	Cross-sectional
Ladera *et al,* 2017	Venezuela	Patients with invasive and in situ BC (April 2014- May 2015)	58.00	Case–control
Lawson *et al,* 2017	Australia	Patients with benign breast biopsies 1-11 years prior to developing BC	56.10	Case–control
Naushad *et al,* 2017	Pakistan	Patients with primary invasive BC (2012 to 2014)	Not specified	Case–control
Gupta *et al,* 2021a	Croatia	Patients with triple-negative BC	62.40	Case–control
Gupta *et al,* 2021b	Qatar	Patients with BC treated surgically (January 2008- December 2019)	55.30	Cross-sectional
Metwally *et al,* 2021	Egypt	Patients with invasive carcinoma treated surgically (2018-2020)	41.30	Case–control
Nagi *et al,* 2021	Lebanon	Patients with invasive ductal carcinomas (2006 and 2016)	52.40	Case–control
Nakhaie *et al,* 2021	Iran	Patients with BC (April 2020 - October 2020)	47.4	Case–control
Fani *et al,* 2023	Iran	Patients with ductal, lobulillar or mucinous BC (2018-2021)	49.50	Case–control
Khasawneh *et al,* 2024	Jordan	Patients with BC treated surgically (2018 and 2022)	55.70	Case–control

Abbreviations: BC, breast cancer.

Table S4. Characteristics of included samples

First author, year	Total BC samples	BC samples included in analysis	Reason for exclusion	HPV+ & EBV+ patients with BC, n (%)	Control samples	HPV+ & EBV+ controls, n (%)	Type of samples	Detection Method	Most prevalent HPV types in EBV+ patients with BC
Aguayo *et al*, 2011	55	46	Samples with negative result in beta-globin test	1/46 (2.17)	0	N/A	FFPE	Conventional and qRT-PCR & ISH	Not specified
Corbex *et al*, 2014	155	123	Samples with negative result in beta-globin test/lack of PEV and TNM status	3/123 (2.44)	0	N/A	FFPE	Multiplex PCR and ISH	HPV16 (in 2 tumors) followed by HPV31 (in 1 tumor)
Peng *et al*, 2014	100	100	N/A	2/100 (2.00)	50	0/50 (0.00)	Tissue biopsies	Primary and nest-PCR & ISH	Samples were screened for HPV18 only
Al Moustafa *et al*, 2016	108	108	N/A	35/108 (32.41)	0	N/A	Paraffin-embedded tissue	PCR and IHC	Not specified
Ladera *et al*, 2017	22	22	N/A	3/22 (13.64)	22	1/22 (4.55)	Tissue biopsies	PCR	Not specified
Lawson *et al*, 2017	27	10	Inadequate tissues or absence of beta globin	2/10 (20.00)	17 (1 sample excluded due to inadequate tissues or absence of beta globin)	1/16 (6.25)	FFPE	Standard and in situ PCR	HPV18 (in 1 tumor) and HPV16/18 coinfection (in 1 tumor)
Naushad *et al*, 2017	250	250	N/A	23/250 (9.20)	115 (15 tissue specimens and 100 blood samples)	0/115 (0.00)	FFPE	Conventional PCR	Not specified
Gupta *et al*, 2021a	70	70	N/A	11/70 (15.71)	16	0/16 (0.00)	FFPE	PCR	Not specified
Gupta *et al*, 2021b	505	74	Samples from males or samples from female patients receiving surgical intervention outside Hamad General Hospital (Qatar) were excluded.	35/74 (47.30)	0	N/A	FFPE	PCR	HPV52 and HPV56
Metwally *et al*, 2021	80	80	N/A	19/80 (23.75)	30	0/30 (0.00)	FFPE, fresh tissue and WBC	PCR	Samples were screened for HPV18 only
Nagi *et al*, 2021	102	102	N/A	30/102 (29.41)	14	0/14 (0.00)	FFPE	PCR and IHC	HPV52 and HPV35
Nakhaie *et al*, 2021	35	35	N/A	2/35 (5.71)	N/A	N/A	Fresh frozen biopsies	Nested-PCR	Not specified
Fani *et al*, 2023	50	50	N/A	0/50 (0.00)	50	0/50 (0.00)	FFPE	PCR	N/A
Khasawneh *et al*, 2024	120	110	Unavailability of molecular subtyping data	2/110 (1.82)	30	0/30 (0.00)	FFPE	RT-PCR	Not specified

Abbreviations: HPV, human papillomavirus; EBV, Epstein–Barr virus; BC, breast cancer; FFPE, formalin-fixed paraffin-embedded; PCR, polymerase chain reaction; ISH, In situ hybridization; IHC: immunohistochemistry.

Table S5. Quality assessment of included articles using Joanna Briggs Institute's checklists.

A. Quality assessment for case-control studies.

Study	i. Group comparability	ii. Group matching	iii. Identification criteria	iv. Methodology of exposure measurement	v. Consistency in exposure measurement	vi. Identification of confounding factors	vii. Handling of confounding factors	viii. Outcome assessment	ix. Exposure period	x. Statistical analysis	Score, n (%)
Peng *et al,* 2014	Y	Y	Y	Y	Y	N	N	Y	UC	Y	7/10 (70)
Ladera *et al,* 2017	Y	Y	Y	Y	Y	Y	N	Y	UC	Y	8/10 (80)
Lawson *et al,* 2017	Y	Y	Y	Y	Y	N	N	Y	UC	NA	6/9 (66.7)
Naushad *et al,* 2017	Y	Y	Y	Y	Y	N	N	Y	UC	Y	7/10 (70)
Gupta *et al,* 2021a	N	Y	Y	Y	Y	N	N	Y	UC	Y	6/10 (60)
Metwally *et al,* 2021	Y	Y	Y	Y	Y	N	N	Y	UC	Y	7/10 (70)
Nagi *et al,* 2021	Y	Y	Y	Y	Y	N	N	Y	UC	Y	7/10 (70)
Nakhaie *et al,* 2021	Y	Y	Y	Y	Y	N	N	Y	UC	Y	7/10 (70)
Fani *et al,* 2023	Y	Y	Y	Y	Y	N	N	Y	UC	Y	7/10 (70)
Khasawneh *et al,* 2024	Y	Y	Y	Y	Y	N	N	Y	UC	Y	7/10 (70)

Joanna Briggs Institute's criteria for case-control studies: i) Were the groups comparable other than the presence of disease in cases or the absence of disease in controls?; ii) Were cases and controls matched appropriately?; iii) Were the same criteria used for identification of cases and controls?; iv) Was exposure measured in a standard, valid and reliable way?; v) Was exposure measured in the same way for cases and controls?; vi) Were confounding factors identified?; vii) Were strategies to deal with confounding factors stated?; viii) Were outcomes assessed in a standard, valid and reliable way for cases and controls?; ix) Was the exposure period of interest long enough to be meaningful?; x) Was appropriate statistical analysis used? Y, Yes; N, No; UC, Unclear; NA, Not Applicable.

B. Quality assessment for analytical cross sectional studies.

Study	i. Inclusion criteria	ii. Participant & study description	iii. Methodology of exposure measurement	iv. Measurement standards	v. Identification of confounding factors	vi. Handling of confounding factors	vii. Outcome assessment	viii. Statistical analysis	Score, n (%)
Aguayo *et al,* 2011	N	Y	Y	Y	N	N	Y	Y	5/8 (62.5)
Corbex *et al,* 2014	Y	Y	Y	Y	Y	Y	Y	Y	8/8 (100)
Al Moustafa *et al,* 2016	N	N	Y	N	N	N	UC	NA	1/7 (14.3)
Gupta *et al,* 2021b	Y	Y	Y	Y	N	N	Y	Y	6/8 (75)

Joanna Briggs Institute's criteria for cross sectional studies: i) Were the criteria for inclusion in the sample clearly defined?; ii) Were the study subjects and the setting described in detail?; iii) Was the exposure measured in a valid and reliable way?; iv) Were objective, standard criteria used for measurement of the condition?; v) Were confounding factors identified?; vi) Were strategies to deal with confounding factors stated?; vii) Were the outcomes measured in a valid and reliable way?; viii) Was appropriate statistical analysis used?

Table S6. Summary of methodological approaches reported for HPV and EBV detection in BC patients

Study	HPV Detection Method	EBV Detection Method
Aguayo et al, 2011	PCR	PCR and ISH
Corbex et al, 2014	PCR	PCR and ISH
Peng et al, 2014	PCR	PCR and ISH
Fimereli et al, 2015	PCR and NGS	IHC and NGS
Al Moustafa et al, 2016	PCR and IHC	PCR and IHC
Shet et al, 2016	PCR	ISH
Ladera et al, 2017	PCR-Hybridization	PCR-Hybridization
Lawson et al, 2017	PCR and NGS	PCR
Naushad et al, 2017	PCR	PCR
Gupta et al, 2021a	PCR	PCR
Gupta et al, 2021b	PCR	PCR
Metwally et al, 2021	PCR	PCR
Nagi et al, 2021	PCR and IHC	PCR and IHC
Nakhaie et al, 2021	PCR	PCR
Oliveira et al, 2022	PCR	IHC and ISH
Fani et al, 2023	PCR	PCR
Khasawneh et al, 2024	PCR	PCR

Abbreviations: PCR: Polymerase chain reaction, ISH: In situ hybridization, NGS: Next generation sequencing, IHC: Immunohistochemistry.
